# Characterisation and comparison of the mucosa-associated bacterial communities across the gastrointestinal tract of stranded green turtles, *Chelonia mydas*

**DOI:** 10.3934/microbiol.2020022

**Published:** 2020-10-19

**Authors:** Mohammad Shamim Ahasan, Thomas B. Waltzek, Leigh Owens, Ellen Ariel

**Affiliations:** 1 College of Public Health, Medical and Veterinary Sciences, James Cook University, Townsville, 4811, Qld, Australia; 2 Faculty of Veterinary and Animal Sciences, Hajee Mohammad Danesh Science and Technology University, Dinajpur 5200, Rangpur, Bangladesh; 3 College of Veterinary Medicine, University of Florida, Gainesville, FL, 32610, USA

**Keywords:** Green sea turtle, *Chelonia mydas*, intestinal microbiology, bacterial diversity, gut microbiota, intestinal microbiome

## Abstract

*Chelonia mydas* are primarily herbivorous long-distance migratory sea turtles that contribute to marine ecosystems. Extensive research has been conducted to restore the populations of green turtles. Little is known about their gut microbiota which plays a vital role in their health. We investigated the mucosa-associated bacterial communities across the gastrointestinal (GI) tract of a total four (3, juvenile and 1, adult) stranded green turtles. Samples taken from four GI regions including oesophagus, stomach, small intestine and large intestine were analysed by high-throughput sequencing targeting hypervariable V1-V3 regions of the bacterial 16S rRNA gene. Bacterial diversity and richness decreased longitudinally along the GI tract from oesophagus to the small intestine of stranded turtles. The large intestine showed a higher bacterial diversity and richness compared to small intestine. The bacterial community of green turtles' GI tract was largely dominated by Firmicutes, Proteobacteria, Actinobacteria, Bacteroidetes and Fusobacteria. Aerobic and facultative anaerobic bacteria prevailed primarily in the oesophagus while anaerobes (*Lachnospiraceae*, *Peptostreptococcaceae* and *Ruminococcaceae*) constituted the bulk of large intestinal microbiota. Firmicutes dominated the GI tract except within the small intestine where Proteobacteria prevailed. At the OTU level, six percent of the total OTUs (>1% relative abundance) were common in all GI regions. This is a comprehensive characterisation of bacterial microbiota across the GI tract in green turtles which will provide a reference for future studies on turtle gut microbiome and their metabolism to improve their health and nutrition during rehabilitation.

## 1. Introduction

Microbial communities inhabiting the gastrointestinal (GI) tract comprise a complex ecosystem and play a vital role in maintaining host physiology, ranging from metabolic activity to host immune homeostasis [1],[2]. They are also involved in many functions that are not encoded in the host‘s DNA [3]. In recent decades, several studies on gut microbiota have confirmed that a balanced gut microbiome is essential for the host's ability to maintain a healthy state. Perturbations in the stability of gut microbial communities dispose the host to pathogenic invasions which may lead to several GI diseases and disorders [4]–[7]. Knowledge of microbial community and population dynamics in symbiosis, as well as in dysbiosis, are essential for developing management strategies for treating GI associated diseases and disorders.

Recent advancement in the molecular biology and computational techniques have empowered the scientific community to explore several influencing factors on the establishment and maintenance of host gut microbiome. Studies revealed that diet is one of the primary drivers of functional capacity within the GI tract, resulting in a convergence of bacterial communities and the phylogenetically un-related host [8],[9]. Besides diet, several host factors such as physiology, gut structure and genetics can also shape gut microbial communities [10],[11]. Studies in terrestrial and aquatic animals revealed that several environmental factors such as habitat, temperature and salinity can influence the microbial community compositions within the GI tract of the host [12]–[14].

To date, large scale research on host gut microbiome has been conducted on several aquatic and terrestrial mammals [9],[15],[16], fishes [17], birds [18]–[20] and to a lesser extent invertebrates [21]–[23]. Few studies have investigated marine reptiles, including the endangered green turtles [24]–[26]. Green sea turtles are a long-distance migrators, hind gut fermenters, which forages primarily on sea grasses [27]. The gut microbiota of green turtles are believed to play a crucial role in gaining energy from their food sources [28],[29]. Moreover, they can also contribute to several other aspects of their health and development of disease, as noted in other animals [15],[30],[31]. Therefore, detailed understanding of resident gut bacterial communities along the digestive tract of green turtles is very important to develop strategies to treat gut associated disorders and restore the host's normal gut microbiome during rehabilitation.

To date, most investigations of the green turtle's gut microbiome have typically involved bacterial identification in cloacal or faecal contents. The faecal bacterial communities of green turtle mostly dominated by bacteria with in the phyla Firmicutes, Bacteroidetes, Proteobacteria and Actinobacteria while the relative abundance of these bacteria may vary at different health [32], age [24],[25], nutrition [33],[34] and environmental conditions [25],[35]. Additionally, bacteria present in faecal contents only represent the overall gut microbiota rather than bacterial communities of specific anatomical regions of the GI tract [36],[37]. Recently, an investigation of two Hawaiian green turtles' gut microbiota was carried out by *in situ* sampling of gut contents from hindguts (caecum, large intestine and rectum) of the GI tracts and reported more than half of the sequences belonged to the phylum Firmicutes, Bacteroidetes and Proteobacteria while no consistent pattern of bacterial diversity across hindgut was recorded [26]. It is hypothesized that microbiota present in faecal or gut contents represent a pool of both resident and transient microbial population in the GI tract [38]. The mucosal microbiota is more likely to be resident and have a higher potential to influence the host than transient microbial populations and is therefore of more interest to intestinal health [39]. In this study, we therefore, aimed to characterise and compare the mucosa-associated bacterial communities across different regions of the GI tract of stranded green turtles using high-throughput sequencing analysis.

## 2. Materials and methods

### 2.1. Target population and sample collection

Stranded green turtles are occasionally cared for in rehabilitation centers until they recover. In this study, samples were taken from a total of four stranded green turtles that were collected from the Whitsunday beach of the central Great Barrier Reef (Table S1). These turtles died shortly (1 to 4 days) after arriving for rehabilitation. During rehabilitation, turtles were offered human grade squid (*Loligo opalescens*), but were not interested in feeding. The freshly dead turtles were kept frozen (below −20 °C) for subsequent post-mortem examination. The metadata, curved carapace length (CCL) and body weight were recorded in accordance to DEHP (Department of Environment and Heritage Protection) standard operating procedures upon the turtle's arrival to the rehabilitation center [41] (Table S1). Turtle 1 was underweight and emaciated. Turtle 2 was determined to have a healed boat-strike scar on the carapace. No visible traumatic injuries were recorded for turtle 3 and turtle 4 however, they were emaciated and infested by barnacles.

During the necropsies, samples were collected from four different regions of the GI tract, including the oesophagus, stomach, small intestine (duodenum) and large intestine (caecum) of the animals. Using a sterile scalpel blade, the luminal cavity of each GI region was opened and the gut contents were separated from the mucosal wall. A deep mucosal scraping sample was collected using a sterile cotton swab rubbing thoroughly against the outer mucosal layer of the gut lumen with minimum contamination by gut contents. Swabs were placed in microfuge tubes, transported on ice within 4–6 hrs directly to the laboratory at James Cook University (Townsville, QLD), and frozen at −80 °C until processing. This study was conducted under permit from James Cook University Animal Ethic Committee (Permit no. A2101), Department of Environment and Heritage Protection Authority (permit no. WISP15015914) and Great Barrier Reef Marine Park Authority (Permit no. G14/37285.1).

### 2.2. Extraction of bacterial DNA, PCR amplification and sequencing

The PowerLyzer® PowerSoil® DNA Isolation Kit was used to extract bacterial nucleic acid from mucosal swab samples following the manufacturer's standard protocol (MO BIO Laboratories, Inc., Carlsbad, CA, USA). Once extracted, the quantity of the extracted DNA was determined using GeneQuant Prospectrophotometer (Amersham Pharmacia Biotech) and stored at −20 °C until used.

Genomic DNA extracted from mucosal swab samples were used as templates for PCR amplification of the hypervariable V1-V3 regions of the bacterial 16S rRNA gene using the forward (27F 5′-AGAGTTTGATCMTGGCTCAG-3′) and reverse (519R 5′-GWATTACCGCGGCKGCTG-3′) primers, and required Illumina flowcell adaptor sequences [42]. The PCR reaction was carried out using an initial denaturation at 95 °C for 7 min, followed by 29 cycles of denaturation at 94 °C for 45 second, primer annealing at 50 °C for 1 minute and extension at 72 °C for 1 minute, with a final elongation at 72 °C for 7 minutes. The AmpliTaq Gold 360 mastermix (Life Technologies, Australia) was used for the primary PCR and a secondary PCR to index the amplicons was performed with TaKaRa Taq DNA Polymerase (Clontech). The resulting amplicons were measured by fluorometry (Invitrogen Picogreen) and normalized. The eqimolar pool was then estimated using qPCR (KAPA), followed by sequencing on the Illumina MiSeq platform (San Diego, CA, USA). Sequencing and library preparation were done by the Australian Genome Research Facility, Brisbane, Australia.

### 2.3. Bioinformatic analysis

All bioinformatics analysis were performed using the software package ‘The Quantitative Insights Into Microbial Ecology (QIIME v1.9.1)’ [42]. A unique barcode was used to label each sample and Illumina sequencing resulted in 17,22,641 paired-end reads from a total of 16 samples. The raw paired-ends Fastq files were quality trimmed (q < 20) to remove low-quality and single end reads using Sickle (version 1.33) (https://github.com/najoshi/sickle). The forward and reverse reads were assembled using PANDAseq Assembler [43]. Using USEARCH (version 8.0.1623), sequences were quality filtered to remove the chimeric reads and full length duplicate sequences [44]. Sequences were then clustered into operational taxonomic units (OTUs) using UCLUST taxonomy assigner in QIIME. To obtain the number of reads in each OTU, reads were mapped back to OTUs with a minimum identity of 97%. The reads that failed to hit the reference database were subsequently clustered as de novo. Using QIIME, taxonomy was assigned using Silva database (version 128, Sep 2016) (https://www.mothur.org/wiki/Silva_reference_files#Release_128). The sequences that were unable to assign to any known taxa were categorized as ‘Unclassified’.

### 2.4. Statistical analyses

A new OTU table was constructed considering OTUs that showed a relative abundance > 1% and samples that retained > 1000 sequence reads. Bacterial count data were then normalised by total sum scaling (TSS) normalization method. The abundance based coverage estimator (ACE), Chao1 and Shannon diversity indices were used to determine bacterial richness and diversity of samples using R software (version 3.3.0) [45]. One-way ANOVA was used to test for differences in the bacterial diversity/richness among different GI regions. Individually based rarefaction curves were constructed to determine whether sampling yielded sufficient OTU coverage to delineate the gut bacterial composition of each turtle. Furthermore, conditional uncovered probability (CUP) and Good's coverage were estimated for each sample in QIIME. Distances between bacterial communities of different samples of different GI regions were calculated using weighted UniFrac distance matric [46] and further visualised by Principle Coordinate Analysis (PCoA) in Calypso [47]. The analysis of similarity (ANOSIM) was performed by applying Bray-Curtis [48] and weighted UniFrac [46] distance matrices to evaluate the association between bacterial communities of different GI regions. The taxa level differences among bacterial communities of different GI regions were assessed by ANOVA and Wilcoxon signed-rank test. A significant p-value (P < 0.05) indicated if the taxa were significantly different between GI regions. P-values were also adjusted for multiple testing by the false discovery rate (FDR) using Benjamini and Hochberg method [49]. Venn diagrams were constructed to visualise the amount of unique and mutually shared gut bacterial communities in different GI regions.

## 3. Results

A total of 703,799 high-quality sequences obtained after assembly and quality filtering were used for downstream analyses. The mean sequence length ranged from 434 to 500 bp (avg. 468.43 bp). Individual samples were covered by an average 43,987 reads (ranging from 15,125 to 75, 952). The highest number of reads was recorded for the oesophageal samples. All sequences were delineated into OTUs with 97% nucleotide sequence identity threshold, where 23,392 unique OTUs were identified in total and 405 OTUs were retained after filtering the low abundant OTUs (relative abundance < 1%) and OTUs that retained above 1,000 sequences (up to 57,928 sequences). The highest number of OTUs were recorded for large intestinal (LI) samples (123.00 ± 29.16) while a lower number of OTUs (64.25 ± 6.49) were in the small intestinal (SI) samples of green turtles (Table 1). The individually based rarefaction curves were constructed that tended to approach saturation plateau in our current sequencing depth (Figure S1). The mean conditional uncovered probability (PE) for different GI region samples ranged from 0.02 to 0.04 (Table 1) and the Good's coverage was > 92% (ranging from 92 to 99%) for all samples, suggesting the presence of sufficient phylotypes for the current analysis although more phylotypes may be recovered by increasing the sequencing depth (Table 1).

**Table 1.** Alpha diversity metrics for the bacterial communities across different regions of the gastrointestinal (GI) tract at an evolutionary distance D = 0.03. Values indicates means and standard errors derived from total samples for each GI regions. ES: Oesophagus, ST: Stomach, SI: Small intestine and LI: Large intestine.

Indices	ES	ST	SI	LI
Sequences	48535.50 ± 10864.05	37154.00 ± 2571.05	24939.25 ± 3098.35	41552.50 ± 10551.54
OTUs	109.25 ± 19.01	114.00 ± 9.14	64.25 ± 6.49	123.00 ± 29.16
Chao 1 index	144.80 ± 22.71	139.86 ± 12.77	87.07 ± 11.33^a^	157.41 ± 31.99
ACE index	148.61 ± 25.40	138.05 ± 13.10	90.62 ± 11.93^b^	157.28 ± 28.99
Shannon index	4.91 ± 0.47	4.33 ± 0.40	2.82 ± 0.63^c^	4.24 ± 1.01
Good's coverage	0.97 ± 0.01	0.97 ± 0.01	0.97 ± 0.01	0.96 ± 0.01
Conditional Uncovered Probability (CUP)				
PE	0.03 ± 0.01	0.02 ± 0.00	0.02 ± 0.01	0.04 ± 0.01
Lower bound	0.004 ± 0.002	0.003 ± 0.001	0.004 ± 0.001	0.006 ± 0.002
Upper bound	0.042 ± 0.016	0.028 ± 0.006	0.040 ± 0.014	0.056 ± 0.017

Note: Superscript ‘^a, b^’ indicate Chao 1 and ACE index of SI were significantly different from ST indices (ANOVA, *P* < 0.05). ‘^c^’ indicates Shannon index of SI was significantly different (ANOVA, *P* < 0.05) from ES and ST.

### 3.1. Assessment of bacteria richness and diversity

The Chao 1 and abundance-based coverage estimators (ACE) were used to evaluate the community richness of each sample from different regions of GI tract (Table 1). Bacterial richness was lower in the small intestinal samples compared to oesophageal, stomach and large intestinal samples. However, no significant difference (*P* > 0.2) was found between observed OTUs and actual OTUs, estimated by Chao 1 and ACE estimators for any GI region. Bacterial diversity as estimated by the Shannon index (SI) revealed significant difference between oesophageal and small intestinal samples (t test, *P* = 0.037). Additionally, the stomach and large intestinal mucosa also harbored a relatively higher level of diverse bacteria than the small intestinal mucosa. However, at this sequencing depth, oesophageal samples showed the highest bacterial diversity (mean SI 4.91 ± 0.47) compared to other samples from different GI regions.

### 3.2. Variation in bacterial microbiomes across the GI tract

**Figure 1.**
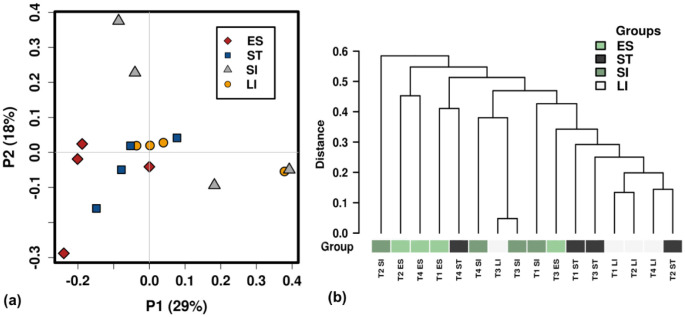
Principle coordinates analysis (PCoA) of the dissimilarity among samples from different regions of gastrointestinal tract of green turtles. (a) PCoA plots and (b) hierarchical dendrograms were constructed using weighted UniFrac distance matric. T1: turtle 1, T2: turtle 2, T3: turtle 3, T4: turtle 4, ES: Oesophagus, ST: Stomach, SI: Small intestine and LI: Large intestine.

Although several bacterial communities exhibited a consistent presence across the length of the green turtles' GI tract, the overall composition of mucosa-associated bacterial microbiome was found to be significantly associated with GI regions (ANOSIM R: 0.28, *P* < 0.05, Bray-Curtis, Figure S2). The strongest association between bacterial microbiomes and GI regions was recorded for the small and large intestinal mucosa-associated bacterial communities (ANOSIM R: 0.469, *P* < 0.05, Bray-Curtis). PCoA plot and hierarchical dendrograms were constructed based on weighted UniFrac distance metric, clearly demonstrated that the majority of large intestinal samples were clustered together and distinctly separated from small intestinal samples although a higher variation was noticed for small intestinal samples (Figure 1). The oesophageal and stomach samples were clustered closely to one another and clearly separated from small intestinal samples.

### 3.3. Comparison of mucosa-associated bacterial communities of different regions of GI tract

**Figure 2.**
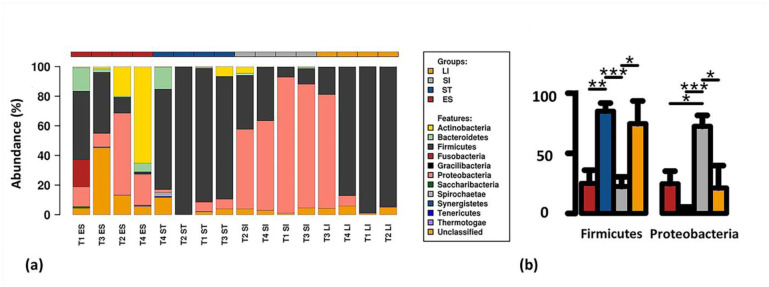
Composition of green turtle gut microbiomes. (a) cumulative abundance (%) of the bacterial communities at phylum level across different regions of the gastrointestinal (GI) tract. (b) Relative abundance of two most dominant phyla across the GI tract. T1: turtle 1, T2: turtle 2, T3: turtle 3, T4: turtle 4, ES: Oesophagus, ST: Stomach, SI: Small intestine and LI: Large intestine. Significantly different regions are shown in barchart using Wilcoxon signed-rank test (* p < 0.05, **: p < 0.01, ***: p < 0.001). Standard error is depicted by error bars.

Phylum-level analysis of the mucosa-associated bacterial communities of green turtle GI tract revealed a total of 29 bacterial phyla, however, majority (17/29) are lower in abundant (<1%). The majority of the bacterial community present within the GI tract belonged to Firmicutes (57.8%) and Proteobacteria (21.3%), while the rest were distributed amongst Actinobacteria (6.4%), Bacteroidetes (3.6%), Fusobacteria (2.4%), Spirochaetae (0.19%), Saccharibacteria (0.16%), and unclassified bacteria (7.9%) (Figure 2a). Other phyla, such as Thermotogae, Tenericutes, Synergistetes, Gracilibacteria, Chloroflexi, comprised <0.25% of total composition. Oesophageal and stomach mucosa harboured the majority of the identified phyla (24 and 22 of the 29), while the lowest number of bacterial phyla were observed in the small intestinal mucosa. Among the phyla identified in this study, only Firmicutes, Proteobacteria and Actinobacteria were present in samples of all GI regions. These phyla were ubiquitously distributed in the GI tract whilst the relative abundance varied between the gut sites. The phylum Firmicutes was significantly abundant in oesophageal (30.3%), stomach (85.1%) and large intestinal (86.5%) mucosa-associated microbial communities, while a drastically lower abundance was observed for the small intestinal bacterial population (Figure 2b). The relative abundance of Proteobacteria was significantly higher (*P* < 0.05) in small intestinal microbial communities than other GI regions (Figure 2b). In summation, Proteobacteria showed an inverse profile to Firmicutes and was the second most abundant phylum across the GI tract. The relative abundance of Actinobacteria, Bacteroidetes and Fusobacteria was higher in oesophageal mucosa-associated bacterial communities compared to other GI regions (Figure 2a).

**Figure 3.**
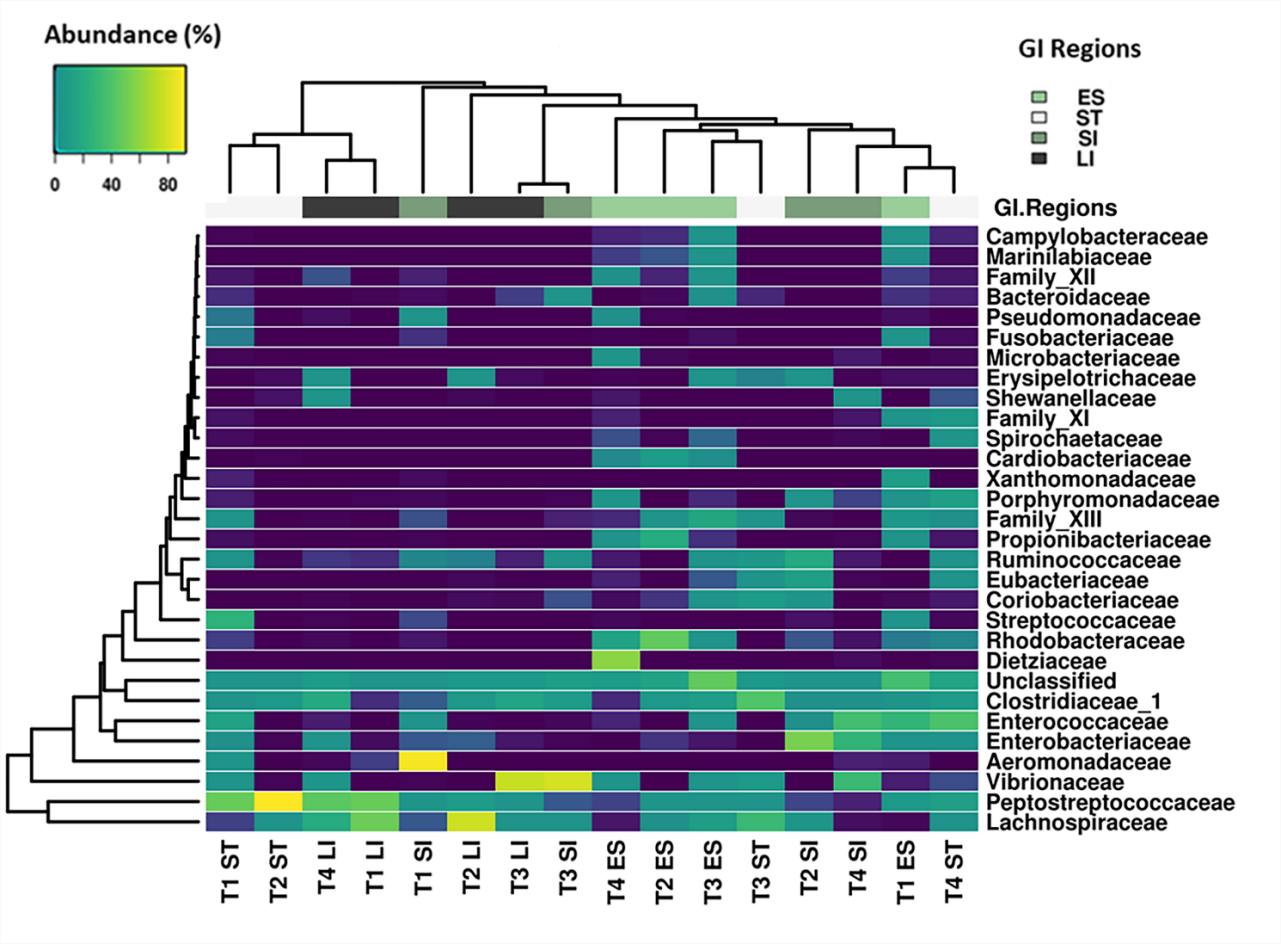
The heatmap indicating relative abundance of major bacteria families across different regions of the gastrointestinal tract of green turtles. ES: Oesophagus, ST: Stomach, SI: Small intestine and LI: Large intestine.

**Figure 4.**
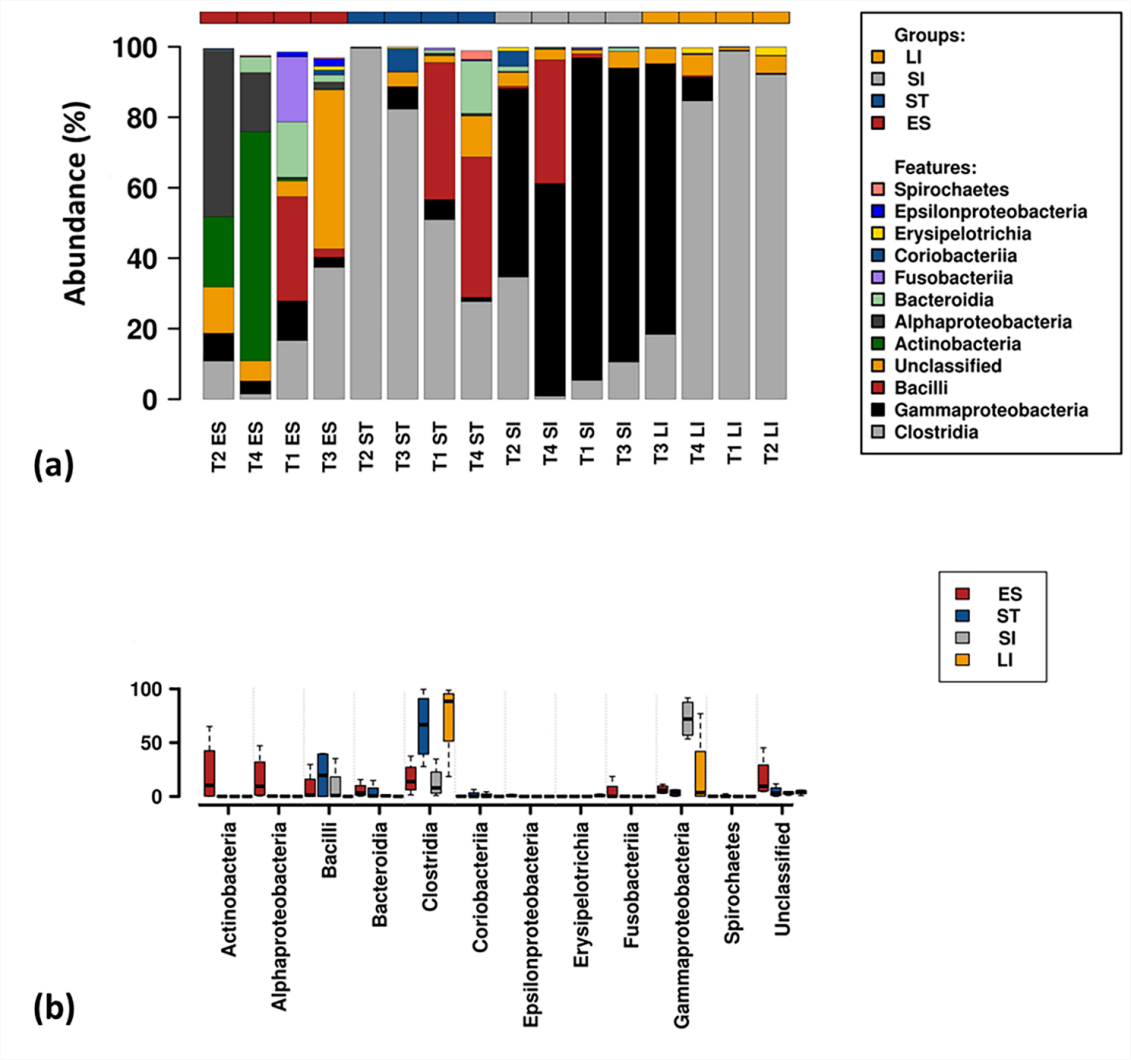
Composition (a) and intra-class variations (b) of most abundant bacteria classes across different regions of the gastrointestinal tract of green turtles. T1: turtle 1, T2: turtle 2, T3: turtle 3, T4: turtle 4, ES: Oesophagus, ST: Stomach, SI: Small intestine and LI: Large intestine.

**Figure 5.**
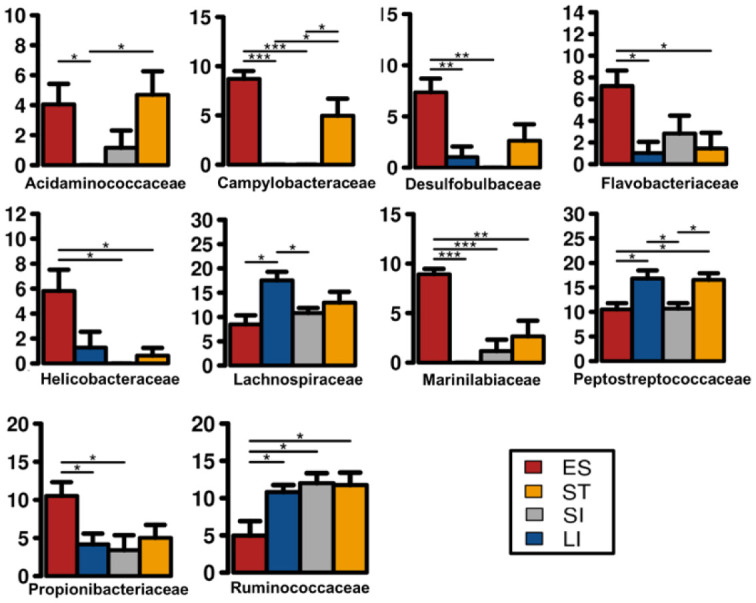
The top 10 bacteria families that were significantly different across the gastrointestinal tract of green turtles. ES: Oesophagus, ST: Stomach, SI: Small intestine and LI: Large intestine. Significantly different regions are shown in barchart using Wilcoxon signed-rank test (* p < 0.05, **: p < 0.01, ***: p < 0.001). Standard error is depicted by error bars.

A total of 190 families were identified from the complete data set. The 30 most abundant families (Figure 3) belonged to 11 different classes that include Clostridia, Gammaproteobacteria, Bacilli, Actinobacteria, Bacteroidia, Alphaproteobacteria, Fusobacteria, Epsilonproteobacteria, Spirochaetes, Coriobacteria and Erysipelotrichia (Figure 4). Clostridia was one of the most abundant bacterial classes in all anatomic regions, except the small intestine, where a significantly lower (*P* < 0.05) abundance of Clostridia was observed. Clostridia was mainly comprised of seven families, *Peptostreptococcaceae*, *Lachnospiraceae*, *Clostridiaceae 1, Ruminococcaceae*, *Eubacteriaceae*, Family XIII and Family XI that accounted for an average 45.6% of the total identified families. Bacteria within the family *Peptostreptococcaceae* were significantly abundant (*P* < 0.007) in stomach and large intestinal samples compared to oesophageal and small intestinal samples (Figure 5). A high abundance (*P* = 0.02) of *Lachnospiraceae* was observed only in large intestinal samples compared to oesophageal and small intestinal samples. Bacteria within the family *Ruminococcaceae* was significantly lower (*P* = 0.02) in the oesophageal mucosa compared to other GI regions. The relative abundance of Gammaproteobacteria was higher in small intestinal samples without any significant difference (*P* = 0.064) to other GI regions. Gammaproteobacteria includes *Aeromonadaceae*, *Vibrionaceae*, *Enterobacteriaceae* and *Xanthomonadaceae*, all of which were dominant in small intestinal samples except *Xanthomonadaceae*, found only in oesophageal samples. The prevalence of Bacilli was lower in the large intestinal mucosa compared to other GI regions. A higher abundance of Bacteroidia (*Porphyromonadaceae* and *Marinilabiliaceae*) was observed within the oesophageal and stomach mucosa-associated bacterial communities (Figure 5). Likewise, Alphaproteobacteria (*Rhodobacteraceae*) and Actinobacteria (*Dietziaceae* and *Propionibacteriaceae*) were absent in large intestinal samples, but were higher in abundance in oesophageal samples. The *Campylobacteraceae*, *Helicobacteraceae*, *Acidaminococcaceae*, *Desulfobulbaceae*, *Flavobacteriaceae* and *Marinilabiaceae* significantly prevailed in the oesophageal mucosa-associated bacterial communities. Our analysis revealed that one or two families represented the majority of OTUs and were highly abundant in the turtles. For example, *Vibrionaceae* 17162, *Aeromonas* 19906, uncultured *Clostridium* sp., *Peptostreptococcaceae* 17846 and *Clostridiaceae* 1 5902 comprised of 5.3%, 4.6%, 4.5%, 4.2%, 3.3%, and 2.0% of the total sequences respectively. At the genus level, 459 taxa were identified across the GI tract of green turtles while more than 35.8% of the total sequences were not identified at the genus level.

**Figure 6.**
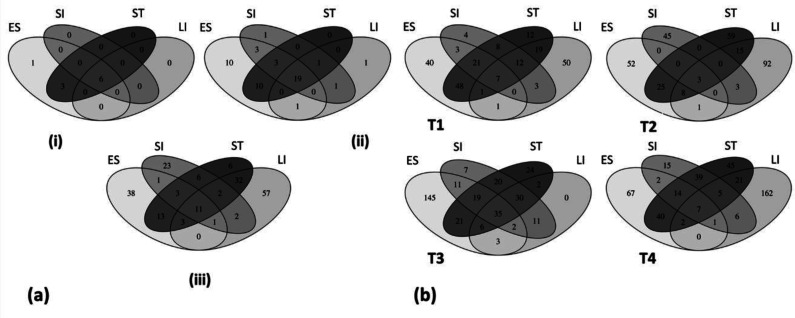
Venn diagrams represent the numbers of total shared and exclusive taxa. (a) Taxa at phylum (i), family (ii), and OTU (iii) level within the different gut regions of green turtles. (b) Taxa at OTU level in each green turtle. T1: turtle 1, T2: turtle 2, T3: turtle 3, T4: turtle 4, ES: Oesophagus, ST: Stomach, SI: Small intestine and LI: Large intestine.

We constructed a Venn diagram to determine the unique and mutually shared bacterial taxa present across the GI tract of green turtles. Our results showed that only 11 (5.6%) of the total OTUs (relative abundance > 1%) were common in all gut regions, while 38 (19.2%), 6 (3%), 23 (11.6%) and 57 (28.6%) OTUs were strictly associated with oesophageal, stomach, small and large intestinal bacterial communities respectively (Figure 6a). Moreover, eight of the 11 common OTUs were within the phylum Firmicutes and three OTUs represented Proteobacteria, Bacteroidetes, and Fusobacteria respectively (Table S2). Sixteen (8.1%) of the total OTUs were common between small intestinal and large intestinal bacterial communities. At the family level, 19 (38%) families were shared among all GI regions and 32 (64%) families were exclusively shared between oesophageal and stomach mucosa-associated microbiomes (Figure 6a). Six of the 10 phyla (relative abundance >1%) were distributed across the GI tract of green turtles while three phyla were exclusively present in oesophageal and stomach mucosa-associated bacterial communities (Figure 6a). It is worth noting that inter-turtle variations were also observed from the phylum to OTU levels of the bacterial communities of green turtles (Figure 6b). Only six of the total (405) OTUs were found in all green turtles, where five represented the phylum Firmicutes.

## 4. Discussion

To date, little is known about the diverse gut microbiota of endangered green turtles [24],[25],[33] in contrast to the extensive knowledge of their ecology, as well as distribution [50],[51]. Knowledge of the microbial community is imperative to understand the host-microbial interaction in both healthy and diseased animals. In this study, we explored the community composition and structure of the mucosa-associated bacterial microbiomes across the length of gastrointestinal (GI) tract of stranded green turtles using high-throughput sequencing and identified an enormous diversity of microbes.

As green turtles are endangered species, it is close to impossible to obtain permits to kill healthy turtles for research purposes in Australia. The samples for this study were therefore collected from turtles that died within one week of entering rehabilitation and without having consumed the food offered. Although the underlying causes of the strandings may have varied between the 4 turtles and thereby influenced the intestinal microbiota in different ways, there was little variation between samples collected in the four regions of the intestinal tract and we therefore feel that is it appropriate to proceed to make general conclusions based on these samples. Gut bacterial diversity decreased across the longitudinal axis of the green turtle GI tract from oesophagus to small intestine while the large intestine showed a higher bacterial diversity compared to the small intestine but less than stomach. This finding is in accordance with findings in terrestrial herbivores, such as cattle, where bacterial diversity was higher in the stomach compared to small intestinal mucosa [15]. The presence of highly diverse bacterial communities in the oesophageal and stomach mucosa could be due to the existence of transient microbiota. Highly diverse bacterial communities in sea water might influence the diversity of bacterial microbiomes in the oesophageal mucosa of green turtles because turtles are continuously ingesting bacteria associated with their food and water [52],[53]. These findings support the theory speculating that the mouth is an access point for gut microbes (highest diversity) with selection for a subgroup of the cumulative diversity appearing as these microbes pass through the GI tract. However, studies in laboratory mice revealed that the microbial diversity in stomach and small intestinal mucosa were similar to the large intestinal mucosa [54]. The presence of a diverse bacterial community in the large intestinal mucosa of green turtles indicates the occurrence of a more complex micro-ecosystem that might be associated with nutrient absorption and assimilation [55] and possibly the availability of oxygen [56]. A similar finding was also observed in the colonic mucosal samples of rat and humans [36].

The GI mucosa-associated microbiota of green turtles were largely dominated by Firmicutes, Proteobacteria, Actinobacteria, Bacteroidetes, Fusobacteria and a considerable number of unclassified bacteria. However, the detailed composition of these phyla at a lower taxonomic level differed notably in different GI regions. Our results demonstrated that Firmicutes were consistently present across different regions of the GI tract mucosa, however, these bacteria were significantly dominant in stomach and large intestine compared to esophagus and small intestine. The higher prevalence of Firmicutes was supported by findings in other studies on green turtle using cloacal swab or faecal contents samples [24],[25],[57] as faecal swab represents the overall gut microbial population of green turtles. A similar finding was also reported by Mcdermid *et. al*., 2020 [26] in Hawaiian green turtles where *in situ* gut contents samples of the hindgut were used for analysis. Additionally, the study suggested to include samples across the GI tract to investigate whether these differences are significant. Members of Firmicutes have been found to be associated with harvesting energy and absorption of nutrients through microbial fermentation of feed components [8],[58]. Studies suggested that the caecum is the initial site for this fermentation activity while little digestion and absorption may take place in the stomach and small intestine [28],[29]. In green turtles, examination of the GI ingesta demonstrated that many of the plant cell materials remained undigested until it reached the caecum where microbial fermentation occurs [28]. Our current study revealed that several families within the phyla Firmicutes, such as *Ruminococcaceae*, *Lachnospiraceae*, and *Peptostreptococcaceae*, had a significant presence in the large intestinal mucosa while *Peptostreptococcaceae* and *Ruminococcaceae* also dominated the stomach mucosa. *Lachnospiraceae* was more abundant than *Ruminococcaceae* in the large intestine, as reported previously by Campos and her colleagues for the excrements of green turtles from Brazil [57] and by Hong and her colleagues for excrement of marine iguanas [59]. The opposite is true in terrestrial herbivores such as cattle [15], and pigs [39]. It is speculated that the composition of cell wall of macroalgae and terrestrial plants may explain the difference [57]. Juvenile green turtles in eastern Australia forage primarily on macroalgae, while adults preferentially consume seagrass [60]. Green turtles sampled in this study were all juveniles and a macroalgael based diet might explain the prevalence of *Lachnospiraceae* over *Ruminococcaceae* in these animals. Bacteria within these families are known to proliferate by hydrolyzing dietary fibre and complex carbohydrates [61] with *Ruminococcaceae* and *Lachnospiraceae* being major butyrate producers that reduce leeky gut syndrome, reduce available oxygen in the gut and prevent pathogenic Proteobacteria from dominating the gut [56]. *Peptostreptococcaceae* can also play a vital role in feed degradation and digestion [62],[63]. However, bacterial community structure and membership within the GI tract can be influenced by factors such as, physiochemical conditions along the GI tract including GI motility, pH and GI secretions. A recent study using humanized mice (germ-free mice colonized with human faecal microbiota) clearly revealed that GI motility can alter the gut microbial composition of the host although it is dependent on diet [64]. Studies in human colonic microbiota demonstrated that a lowering pH from substrate fermentation in the large intestine may increase butyrate production and populations of butyrate-producing bacteria while at the same time inhibiting the Bacteroides spp. [65]. The availability of different substrates and nutrients along the GI tract may also promote distinct bacterial populations in different GI regions of the host [66]. Moreover, the availability of oxygen in different GI regions can also play a vital role in gut bacterial compositions [67]. Oxygen availability in the oesophagus and stomach was higher, therefore, the aerobic and facultative bacteria including *Propionibacteriaceae*, *Desulfobulbaceae*, *Marinilabiaceae*, *Campylobacteriaceae*, *Helicobacteriaceae*, *Flavobacteriaceae* were significantly enriched in oesophageal and stomach mucosa. In contrast, in absence of oxygen, anaerobes or oxygen sensitive bacterial communities such as *Lachnospiraceae*, *Clostridiaceae*, *Ruminococcaceae* were enriched in the large intestinal mucosa of GI tract.

Our results demonstrate that bacteria within the phylum Proteobacteria was significantly less abundant in oesophageal, stomach and large intestinal mucosa while its prevalence was higher in small intestinal mucosa of the GI tract. The present findings are in support to the findings of other study where bacteria within the phylum Proteobacteria recorded less abundant compared to members of Firmicutes and Bacteroidetes in hindguts (caecum, colon and rectum) of Hawaiian green turtles [26]. In this study, the abundance of Proteobacteria in the small intestinal mucosa was largely due to a higher prevalence of the bacteria belonging to the class Gammaproteobacteria, which includes the *Vibrionaceae*, *Enterobacteriaceae* and *Aeromonadaceae*. The majority of these bacteria are known to be facultative anaerobes and multiply at neutral to slightly basic pH (~7.4) [68]. However, previous studies in green turtles revealed a fairly acidic pH (5.5–6) in the large intestinal contents (different from the other reptiles) and a very acidic pH (3.85–4) in the stomach contents [28]. In contrast, the small intestine exhibited a fairly neutral pH (7.0) which provides a suitable environment for the occurrence of Gammaproteobacteria [28],[68]. Gammaproteobacteria, a physiologically and metabolically diverse class, can play an important role in preparing the gut for successive colonization by strict anaerobes through utilizing oxygen, changing the gut pH, producing CO2 and nutrients [69],[70]. However, an over colonization of these opportunistic bacteria (*Vibrionaceae* and *Enterobacteriaceae*) common markers of dysbiosis [56] within the small intestinal mucosa may competitively exclude the other normal flora and result in decreased bacterial diversity in the small intestinal mucosa [71],[72]. Further investigation is suggested to explore these phenomena.

Actinobacteria was the third most abundant phylum in the oesophageal mucosa. However, they were very low in other GI regions. The higher prevalence of Actinobacteria in the oesophageal mucosa was mainly due to higher abundance of bacteria in the *Dietziaceae* and *Propionibacteriaceae*. The members of *Dietziaceae* are characterized by the presence of mycolic acids [73]. They are mainly chemoorganotrophic and exhibit an oxidation type of metabolism. However, the diversity of the metabolic activities of different members of this family are still unknown which demand better genetic tools to characterize and exploit their diverse functions within the GI tract of animals [73]. Bacteria within the *Propionibacteriaceae* are well recognized, normal inhabitants of the GI tract for both humans and animals [74],[75]. The pathogenic potential for many Propionibacteria, such as *Propionibacterium* or *Eubacterium*, have been claimed for many years and later on, recognized as opportunistic rather than obligate pathogens [74]. These bacteria are often encountered in multiple infection processes where it is difficult to determine which component of the mixed microbial community is a true pathogen and which is only a ‘hanger-on’ [74]. Furthermore, in this study, bacteria within the phylum Bacteroidetes were less abundant in the small and large intestinal mucosa while it was prominent in oesophageal and stomach mucosa and composed of mainly *Porphyromonadaceae* known members of the buccal cavity associated with the salivary biome [76]. Bacteria within the *Porphyromonadaceae* were also observed in previous studies on cloacal swabs of green turtles [25]. However, in this study, the reason for lower abundance of Bacteroidetes in the small and large intestinal mucosa is still unclear because the majority of food fermentation of green turtles usually takes place in the caecum [28] and Bacteroidetes species are well known to degrade a variety of plant polysaccharides including pectin, xylan, galactomannan, arabinogalactan, alginate and glucomannan [77]–[79] and are major butyrate producers. Therefore, further research is recommended to explore the exact reasons of the low level of Bacteroidetes prevalence in small and large intestinal mucosa.

Furthermore, it is worth noting that the predominance of different phyla in study samples were largely due to the dominance of a small number of distinct OTUs. The top 10 most abundant OTUs, within each GI region, comprised 70.2%, 87.1% and 77.9% of the total resident microbiomes in stomach, small intestinal and large intestinal mucosa respectively. Additionally, four of the top 10 most abundant OTUs of stomach and large intestinal mucosa were common and accounted for 38.4% and 50.4% of the total OTUs. This finding indicates the possibility of similar mucosa-associated microbiota between the stomach and large intestine of green turtles. However, further investigation is suggested to determine the core mucosa-associated gut bacterial community of healthy green turtles.

## 5. Conclusion

We have generated a comprehensive landscape of green turtle bacterial microbiomes across the GI tract in stranded turtles. Our results reveal that mucosa-associated bacterial communities of various compositions occupied different regions of the GI tract. The oesophagus and large intestine exhibit the highest bacterial diversity compared to stomach and small intestine of green turtles. This finding supports the recognized notion that different anatomic regions of the GI tract have their own physiochemical conditions such pH and oxygen, which exert selective pressures on the bacterial communities and play an important role in shaping the microbiota of the GI tract. Interestingly, our study identified several members of opportunistic bacteria across the GI tract which might be overrepresented in stranded turtles. Our results also indicate that faecal samples can only represent the overall bacterial communities rather than microbiota of specific regions of the GI tract. It is therefore advisable to be cautious when interpreting results of faecal samples when investigating gut-associated disease in turtles. Our study provides a helpful reference for further detailed investigation of healthy sea turtle gut microbiomes and their metabolic functions to improve their health and nutrition during rehabilitation.
