# Supplementary material for: Characterisation and comparison of the mucosa-associated bacterial communities across the gastrointestinal tract of stranded green turtles, Chelonia mydas
Source: AIMS Microbiol. 2020 Oct 19;6(4):361–78. doi: 10.3934/microbiol.2020022 (PMC7755585; doi:10.3934/microbiol.2020022)
Supplement: Supplementary file 1 [file microbiol-06-04-022-s001.pdf]

AIMS Microbiology

AIMS Microbiology, 6 (4): 361–378.
DOI: 10.3934/microbiol.2020022
Received: 18 July 2020
Accepted: 08 October 2020
Published: 19 October 2020

http://www.aimspress.com/journal/microbiology

Research article

Characterisation and comparison of the mucosa-associated bacterial communities across the gastrointestinal tract of stranded green turtles, *Chelonia mydas*

Mohammad Shamim Ahasan[1,2,*], Thomas B. Waltzek[3], Leigh Owens[1] and Ellen Ariel[1]

[1] College of Public Health, Medical and Veterinary Sciences, James Cook University, Townsville, 4811, Qld, Australia
[2] Faculty of Veterinary and Animal Sciences, Hajee Mohammad Danesh Science and Technology University, Dinajpur 5200, Rangpur, Bangladesh
[3] College of Veterinary Medicine, University of Florida, Gainesville, FL, 32610, USA

* **Correspondence:** Email: mdshamim.ahasan@my.jcu.edu.au; Tel: +61469851186.

Supplementary Tables

Table S1. Identification number, age-class and morphometric data of each green turtle at arrival. T1: turtle 1, T2: turtle 2, T3: turtle 3 and T4: turtle 4.

Details	T 1	T 2	T 3	T 4
Identification number	QA54944	QA56724	QA56888	QA56344
Location	Whitsunday Beach, QLD	Whitsunday Beach, QLD	Whitsunday Beach, QLD	Whitsunday Beach, QLD
Age-class	Juvenile	Juvenile	Juvenile	Adult
Body weight (kg)	6.1	8.2	12	100
CCL (cm)	48	48.2	52.3	102

Table S2. The abundance of 11 shared OTUs across the gastrointestinal tract of green turtles. ES: Oesophagus, ST: Stomach, SI: Small intestine and LI: Large intestine.

Operational taxonomic units (OTU)	Cumulative abundance (%)			
	ES	ST	SI	LI
Proteobacteria__f__Vibrionaceae_17162	0.67	1.515	23.023	18.348
Fusobacteria__o__Fusobacteriales_19186	4.04	0.085	0.07	0.01
Firmicutes__g__Terrisporobacter_6106	0.05	0.775	0.008	0.09
Firmicutes__g__s__uncultured_Clostridium_sp.__s___9532	0.775	7.345	0.298	6.17
Firmicutes__g__s__uncultured_bacterium__s___808	1.165	6.487	0.055	23.76
Firmicutes__g__s__uncultured_bacterium__s___20735	0.202	10.502	0.058	3.367
Firmicutes__g__Clostridium_sensu_stricto_1_14141	0.08	0.178	0.082	0.168
Firmicutes__g__Ambiguous_taxa__s___8680	5.46	2.282	0.158	0.012
Firmicutes__f__Peptostreptococcaceae_17846	0.922	10.912	0.62	2.203
Firmicutes__f__Clostridiaceae_1_7975	0.842	0.64	0.155	3.635
Cyanobacteria__c__Chloroplast_22627	0.022	0.028	0.188	0.04
Bacteroidetes__g__s__uncultured_bacterium__s___13932	0.67	1.515	23.023	18.348

Supplementary Figures

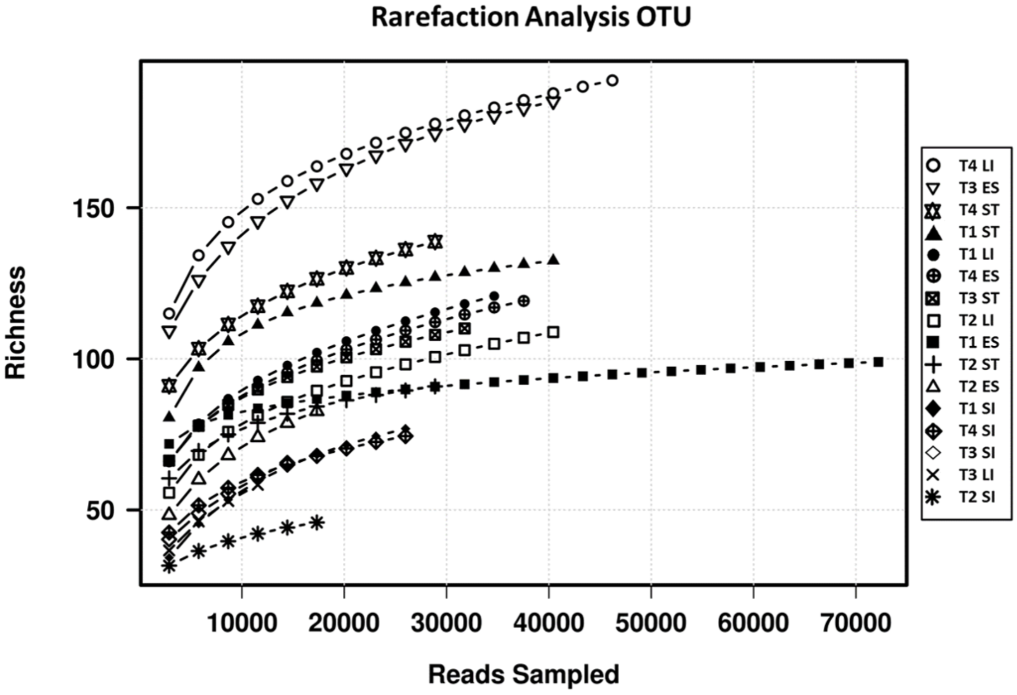

Figure S1. Rarefaction analysis based on operational taxonomic unit (OTUs) (3% divergence) in the samples from different regions of the gastrointestinal tract of green turtles. ES: Oesophagus, ST: Stomach, SI: Small intestine and LI: Large intestine.

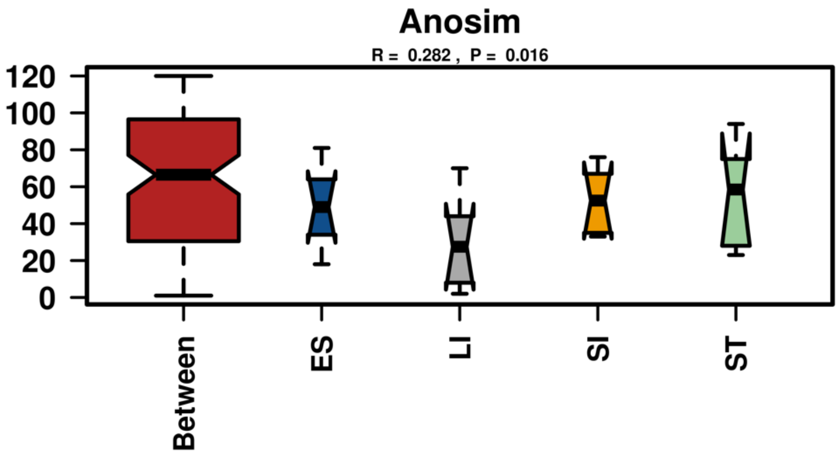

Figure S2. Analysis of Similarity (ANOSIM) in the samples from different regions of the gastrointestinal tract of green turtles using weighted UniFrac distance metric at OTU level.

AIMS Press

© 2020 the Author(s), licensee AIMS Press. This is an open access article distributed under the terms of the Creative Commons Attribution License (http://creativecommons.org/licenses/by/4.0)
